# Assessment of On-Board and Laboratory Gas Measurement Systems for Future Heavy-Duty Emissions Regulations

**DOI:** 10.3390/ijerph19106199

**Published:** 2022-05-19

**Authors:** Barouch Giechaskiel, Tobias Jakobsson, Hua Lu Karlsson, M. Yusuf Khan, Linus Kronlund, Yoshinori Otsuki, Jürgen Bredenbeck, Stefan Handler-Matejka

**Affiliations:** 1 European Commission, Joint Research Centre (JRC), 21027 Ispra, Italy; 2 Scania, CV AB, 15187 Södertälje, Sweden; tobias.jakobsson@scania.com (T.J.); hua.lukarlsson@scania.com (H.L.K.); 3 Cummins Inc., Columbus, IN 47201, USA; yusuf.khan@cummins.com; 4 Volvo GTT, SE-405 08 Göteborg, Sweden; linus.kronlund@volvo.com; 5 Horiba Europe GmbH, 61440 Oberursel, Germany; yoshinori.otsuki@horiba.com; 6 A&D Europe GmbH, 64295 Darmstadt, Germany; bredenbeck@aanddeurope.com; 7 IAG Prüfstandstechnik GmbH, A-2722 Weikersdorf, Austria; s.handler@iag-ng.at

**Keywords:** air pollution, engine emissions, NH_3_, N_2_O, PEMS, portable FTIR, QCL, uncertainty

## Abstract

Road transport contributes significantly to air pollution in cities. Regulations across the globe continuously reduce the limits that vehicles need to respect during their lifetimes. Furthermore, more pollutants are being subject to control with new regulations and, most important, testing tends to be done under real-world conditions on the road. In this study, various portable systems were compared with laboratory-grade equipment with a wide range of emissions, focusing on the lower end, where the measurement uncertainty of the instruments is crucial for the determination of emission limits. The engines were diesel- and compressed natural gas (CNG)-fueled. The results were promising, with relatively small differences between portable emissions measurement systems (PEMSs), portable Fourier transform infrared (FTIR) and quantum cascade laser infrared (QCL-IR) spectrometers, and the respective laboratory-grade analyzers based on chemiluminescence detection (CLD), non-dispersive infrared (NDIR), and FTIR principles. The results also highlighted the need for strict technical regulations regarding accuracy and drift for low emission limits in future.

## 1. Introduction

The European Union (EU) aims for climate neutrality by 2050. The European Commission’s trajectory to zero-emission mobility requires the average CO_2_ emissions of new cars to come down by 55% in 2030 and 100% in 2035 compared to 2021 levels [1]. For heavy-duty vehicles, the EU has set percentage-based CO_2_ reduction goals of 15% and 30% for the years 2025 and 2030 compared to 2019/2020 emissions levels (Regulation (EU) 2019/1242). Even with the new CO_2_ proposal, vehicles with internal combustion engines will remain part of the fleet for 20 or more years [2]. For heavy-duty vehicles, this period might be longer [3]. For this reason, research on heavy-duty vehicle emissions is ongoing [4,5].

Road transport still contributes to air pollution in cities [6,7]. Worldwide policies aim to reduce emissions from internal combustion engines [8]. The European Commission is also working on a Euro 7/VII (light/heavy-duty) proposal to further reduce air pollutant emissions, updating the latest Euro 6/VI standards applicable since 2013/2014. Furthermore, there are discussions underway to add some currently non-regulated pollutants that have increased with the introduction of new after-treatment devices. For example, N_2_O increased with NO_x_ abatement technologies [9,10,11] and NH_3_ with three-way catalysts [12,13]. NO_x_, N_2_O, and NH_3_ have impacts on human health, climate change, and the environment and contribute to particle formation in the atmosphere [14,15,16]. The preparatory work for the future Euro 7/VII standards has been followed and discussed with experts from the industry, non-governmental organizations (NGOs), academia, and Member States in the Advisory Group on Vehicle Emission Standards (AGVES) [17]. The basis of the new standards will be measurements performed on the road and not in the laboratory, as was the case for all previous emission limits. Therefore, there will be no need to apply conformity factors whose goal was to “translate” the measurements made on the road to those made in the laboratory. However, representative measurement uncertainties should be included in the future limits.

Measurements of vehicle emissions on the road with portable emission measurement systems (PEMSs) is a common practice [18,19,20,21]. A lot of work has been done on PEMS measurement uncertainty for the regulated pollutants and particles [22,23,24,25]. Uncertainty can be estimated by combining the uncertainty of the PEMS components that are needed for the calculation of emissions (i.e., analyzer, exhaust flow, work), their drift, the uncertainty introduced by second-by-second measurement (dynamicity, time alignment), and the impact of the boundary conditions (ambient temperature, altitude) on the instrument’s response. It appears that Euro VII standards will include measurements of more pollutants, such as N_2_O and NH_3_, on the road. Various principles can be used to measure these gases. For example, NH_3_ and N_2_O can be measured with Fourier transform infrared (FTIR) or quantum cascade laser infrared (QCL-IR) [26,27] systems. Some studies have demonstrated the feasibility of measuring these pollutants with both light-duty [28,29] and heavy-duty vehicles [30,31]. The uncertainty, however, has not been thoroughly discussed. A few studies have compared various instruments in the laboratory [32,33,34], and even fewer comparisons have been made on the road [35,36]. A review summarized all studies that assessed FTIRs in the lab and on the road [26]: one key conclusion of the review was that there is still a need for more studies, in particular, studies conducted with portable systems, and, most importantly, at the low levels expected for future Euro VII limits. Reviews or studies of instruments using other principles are lacking. For this reason, the European Automobile Manufacturers’ Association (ACEA) in collaboration with the Joint Research Centre (JRC) of the European Commission organized a measurement campaign to test portable systems for vehicle exhaust gases.

The aim of this paper was to assess various principles of measurement of exhaust gases, with a special focus on portable systems and low emission levels. The results of this study could be used by researchers when assessing low-emission vehicles or by legislators when setting future limits.

## 2. Materials and Methods

### 2.1. Setup

**Figure 1:**
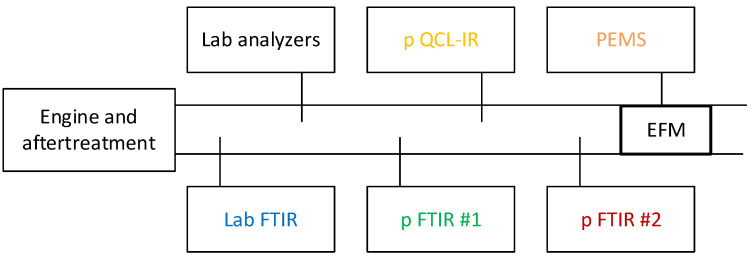
Schematic of experimental setup. EFM = exhaust flow meter; FTIR = Fourier transform infrared; p = portable; PEMS = portable emissions measurement system; QCL-IR = quantum cascade laser infrared.

Five engines were tested from the end of April until the beginning of September 2021 at the facilities of the original equipment manufacturers (OEMs) in Europe, but no engine was circulated. The engines were at the latest regulation stage (Euro VI step E): the diesel engines had diesel oxidation catalysts, diesel particulate filters, selective catalytic reduction for NO_x_, ammonia slip catalysts, and the compressed natural gas (CNG) engine had a three-way catalyst. A typical setup is shown in Figure 1. Four portable systems (PEMS, portable QCL-IR, portable FTIR #1, portable FTIR #2) were compared with laboratory systems (laboratory FTIR, reference gas analyzers). Not all instruments were used with all engines (see details in Table 1). Each laboratory used its own equipment. The only exception was the portable FTIR #2; the same device was used for engines D1 and CNG. The PEMSs were connected to their own exhaust flow meters (EFMs). The exhaust flow measured by the EFM was compared with the exhaust flow measured by the laboratory (intake air plus fuel flow). In one case, on-board diagnostics (OBD) connection was available and the work calculated from the OBD parameters could be compared with the work measured by the engine dynamometer.

### 2.2. Instrumentation

The emissions of each engine were measured with a set of instruments. The reference instruments and the PEMS were different for each engine. The instruments are described in the next paragraphs.

The gaseous pollutants were measured from the tailpipe in real time with the following analyzers: AMA i60 from AVL (Graz, Austria) or MEXA-ONE from Horiba (Kyoto, Japan). The principle of operation of the analyzers was: non-dispersive infrared detection (NDIR) for CO and CO_2_, chemiluminescence detection (CLD) for NO_x_, and hot- (191 °C) flame ionization detection for total hydrocarbons and methane. The gas analyzers had different calibration ranges and the most appropriate was used during the tests, depending on the measured concentration.

The PEMS was the OBS-ONE from Horiba (Kyoto, Japan), which measured CO_2_ and carbon monoxide CO with heated NDIR [37] and NO_x_ with heated CLD [38]. Appropriate size exhaust flow meters (EFMs) were used depending on the size of the engine.

The portable quantum cascade laser infrared (QCL-IR) was the OBS-ONE-XL from Horiba [39], which measured NH_3_ and N_2_O based on the infrared laser absorption modulation (IRLAM) technique [40]. It was connected to the tailpipe with a 6 m polytetrafluoroethylene (PTFE) line heated at 113°C. It used a QCL as a light source modulating its wavelength around the absorption peaks of the target compounds (around 7.8 μm for N_2_O and 10.1 μm for NH_3_). The absorption signal was detected with a non-cooled InAsSb photovoltaic detector. The device’s measurement ranges were 0–1000 ppm for N_2_O and 0–2000 ppm for NH_3_. The LoD (two standard deviations) was <0.3 ppm for N_2_O and <0.7 ppm for NH_3_. The flow rate was 4 L/min; the rise time was <2.5 s.

The laboratory FTIR spectrometer was the AVL Sesam with a Nicolet Antaris IGS Analyzer—Thermo Electron Scientific Instruments LLC (Madison, WI, USA). The instrument was connected to the sampling point with a 6 m heated polytetrafluoroethylene (PTFE) sampling line (191 °C). The analyzer included a Michelson interferometer (spectral resolution: 0.5 cm^−1^, spectral range: 600–3500 cm^−1^), a liquid nitrogen-cooled mercury cadmium telluride (MCT) detector, a multi-path gas cell with 2 m of optical path with a working pressure of 860 hPa, and a downstream sampling pump (typically 8 L/min flow rate). The compounds of interest for this study were NH_3_, N_2_O, NO_x_, and CO_2_. NO_x_ was determined as the sum of NO and NO_2_.

The portable FTIR #1 spectrometer was the OFS from IAG (Weikersdorf, Austria). The instrument was connected to the sampling point with a 6 m (PTFE) heated sampling line (191 °C). The analyzer included a Michelson interferometer (spectral resolution: 0.5 cm^−1^, spectral range: 600–3500 cm^−1^), a liquid nitrogen-cooled MCT detector, a 70 mL multi-path gas cell with 5.1 m of optical path with a working pressure of 860 hPa, and a downstream sampling pump (10 L/min flow rate). 

The portable FTIR #2 spectrometer was the BOB-1000FT from A&D (Darmstadt, Germany). The instrument was connected to the sampling point with a 6 m (PTFE) sampling line (191 °C). The analyzer included a Michelson interferometer (spectral resolution: 0.5 cm^−1^, spectral range: 400–7000 cm^−1^), a liquid nitrogen-cooled MCT detector, a 200 mL multi-path gas cell with 5.1 m of optical path with vacuum working pressure (850 hPa), and a downstream sampling pump (10 L/min flow rate).

Some of the technical characteristics of the instruments are summarized in Table 2. The instruments fulfilled the technical requirements of the United Nations Economic Commission for Europe (UNECE) Regulation 49 for gas analyzers. The most relevant for this study are:

- Linearity requirements: slope 0.99–1.01, R^2^ ≥ 0.998, SEE (standard error of estimate) ≤ 1% max, offset ≤ 0.5% max;
- Accuracy: ±2% of reading or ±0.3% of full scale (whichever is larger). For NH_3_ this requirement is ±3% of reading or 2 ppm whichever is larger);
- Limit of detection: no requirements, except for NH_3_ (<2 ppm). Typically, it is around 1–2 ppm for most gases.

All instruments were connected to the automation system, where the main signals were recorded with 10 Hz frequency. For some instruments, this meant that no error codes or other secondary information was recorded (e.g., temperatures, flows, etc.). All results were included in the analysis.

### 2.3. Test Protocol

The test cycles were the cold and hot start WHTC (world harmonized transient cycle), hot start WHSC (world harmonized steady state cycle) [41] and cold and hot ISC (in-service conformity)-like cycles. The ISC cycles were approximately 3 h long and included urban, rural, and motorway-like conditions. The tests with the diesel engines included exhaust gas with and without the crankcase ventilation connected to the tailpipe, with and without urea injection, and active regenerations. These tests were targeting different levels of particle number emissions and will be the subject of a future publication. Nevertheless, they impacted the gaseous pollutants in some cases. In the presentation of the results, no differentiation is made, unless there is a specific test that needs to be discussed. The reason is that the aim of the paper is the comparison of the instruments and not the absolute emission levels of the engines.

Zero and span adjustments were performed at the beginning of each test for the laboratory analyzers, while no zero and span correction was applied to the FTIRs. The PEMS and the p FTIR were calibrated only in the morning and after the lunch break in order to simulate long on-road tests (>2 h).

### 2.4. Calculations

For each gas (CO_2_, NO_x_, NH_3_, N_2_O) the following equation was used to calculate the gas emissions per cycle work *E_gas_* (g/kWh):

(1) $$E_{gas}=\frac{m_{gas}}{W}=\frac{u_{gas}\sum c_{gas,i}q_{i}/f}{W}$$

where *W* (kWh) is the cycle work, *f* (Hz) is the data sampling rate, *q_i_* (kg/s) is the instantaneous exhaust mass flow, *c_gas,i_* (ppm) is the instantaneous concentration of the gas, and *u_gas_* (−) is a density ratio and units conversion constant. For NO_x_, it is 0.001586 (Diesel) or 0.001621 (CNG) (Table 5 of UNECE Regulation 49). No other correction was applied (e.g., zero/span drift or detection limit). For one instrument (PEMS), the results are presented with and without drift correction in order to demonstrate the effect.

As the instruments were measuring simultaneously, for each test, the differences compared to the reference laboratory analyzer were calculated. For the pollutants, the differences were calculated using the concentrations (ppm) or the final emission rates (g/kWh), using the same flow and work for all instruments, after proper time alignment. Thus, the uncertainty of the flow and work had a minimum impact on the comparisons of the instruments. Nevertheless, the differences between the instruments using concentrations or final emission rates were quite close to each other, except at very low concentrations and with the offset of one of the instruments (Appendix A). 

Appendix A also discusses other uncertainties due to time misalignment and response of the instruments. The uncertainties of flow and work were calculated separately.

## 3. Results

The results are presented separately for the main parameters that are needed for the calculation of emissions (pollutant concentrations, exhaust flow, cycle work).

### 3.1. NO_x_

**Figure 2:**
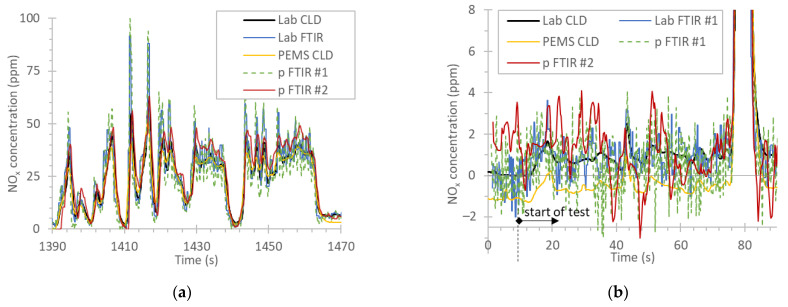
Real-time NO_x_ concentrations of the diesel engine D1: (**a**) part of a typical WHTC; (**b**) first seconds of a WHTC. Reduced scale to focus on the zero levels of the instruments. CLD = chemiluminescence detection; FTIR = Fourier transform infrared; p = portable; PEMS = portable emissions measurement system; WHTC = world harmonized transient cycle.

Figure 2a plots part of a WHTC test where the total cycle NO_x_ emissions were around 70 mg/kWh and the instruments agreed within ±15% on the final emission rate. The NO_x_ concentrations ranged from 0 to 100 ppm, depending on the engine operation point and the after-treatment NO_x_ removal efficiency. The reference instrument used the 200 ppm range. As the real time signals show, in general, the concentrations indicated by all instruments were on top of each other, with small differences due to their differences in response times. The laboratory FTIR and portable FTIR #1 had higher spikes than the other instruments.

Figure 2b plots the first seconds of a test where the NO_x_ emissions were low in order to focus on the background levels of the instruments. Appropriate zeroing of an instrument, typically before the test, reduces any offsets and the signal typically oscillates around zero. Negative values are possible depending on the electronics and sensitivity of the instrument. The emissions of the complete WHTC were around 100 mg/kWh. The noisier pattern of the FTIRs has to do with the high sampling frequency (5 Hz). The zero levels at the beginning of the test were ±0.2 ppm for the laboratory reference CLD, laboratory FTIR, and portable FTIR #1. One point that needs to be highlighted is the −1 ppm offset of the PEMS. The PEMS was not zeroed between the tests to simulate an on-road test of long duration (>2 h) and this WHTC was the last of the day. The −1 ppm offset, even after the linear correction which is allowed in the regulation (i.e., 0.5 ppm), resulted in a 15% underestimation of emissions due to the −15 mg/kWh zero offset. The portable FTIR #2 had an offset of less than 2 ppm (1.5 ppm at the end of the test), which, however, resulted in 15% higher emissions at the end of the test.

**Figure 3:**
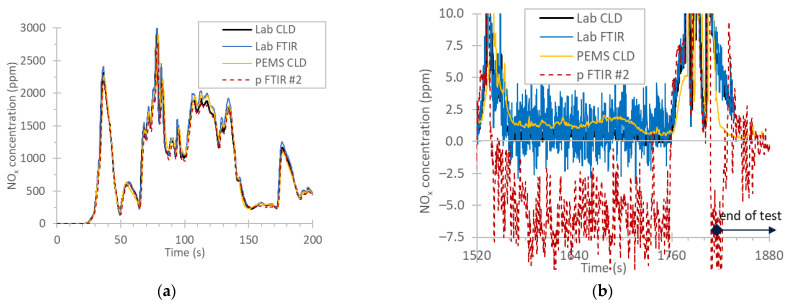
Real time NO_x_ concentrations of the CNG engine: (**a**) first seconds of a cold start WHTC; (**b**) last seconds of a hot start WHTC with reduced scale to low concentrations. CLD = chemiluminescence detection; CNG = compressed natural gas engine; FTIR = Fourier transform infrared; p = portable; PEMS = portable emissions measurement system; WHTC = world harmonized transient cycle.

Figure 3a plots the first seconds of a cold start WHTC with the CNG engine. The NO_x_ concentrations reached up to 3000 ppm; still, the agreement of all instruments was very good, with differences <10%.

Figure 3b plots the last seconds of a hot WHTC, focusing on the zero levels. While most instruments measured around 1 ppm of NO_x_, the portable FTIR #2 measured −6 ppm. After the end of the cycle (time after 1800 s) the portable FTIR #2 returned to 0 ppm. Even though there were no recordings for the other instruments, it is expected that they would come back to <1 ppm. The −6 ppm “wrong” quantification of the portable FTIR #2 could be due to water interference. Although this negative value was not important for the cold start cycles (differences from the reference around −5% for emissions of around 1000 mg/kWh), it resulted in relatively large differences in hot cycles (−25%) for emissions around 150 mg/kWh.

**Figure 4:**
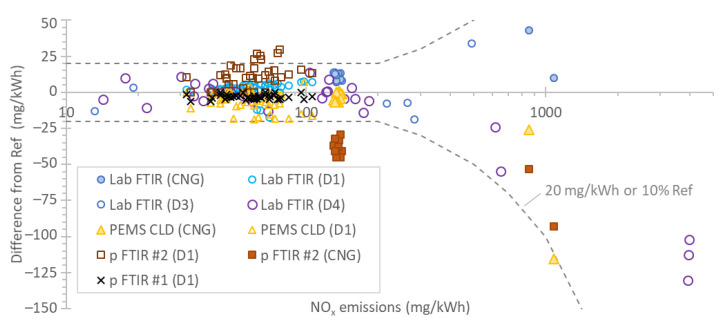
Differences of laboratory (Lab) or portable (p) FTIRs and the PEMS with respect to the laboratory real-time reference CLD analyzers. CLD = chemiluminescence detection; CNG = compressed natural gas engine; D = diesel engine; FTIR = Fourier transform infrared; p = portable; PEMS = portable emissions measurement system.

Figure 4 plots the absolute final emission differences from the reference laboratory CLD analyzers for each instrument as a function of emission levels. All tests performed with all engines are included. Each point is a test cycle and the emissions span from 10 mg/kWh up to 4000 mg/kWh. It should be recalled that the tests include cold starts, regenerations, or no urea injection. In blue are the results for the laboratory FTIRs, in yellow those for the PEMS, in brown those for the portable FTIR #2, and in black those for the portable FTIR #1.

In general, the emissions were within ±20 mg/kWh or within ±10% of the reference laboratory CLD (whichever was larger). The only exception is the portable FTIR #2: the cloud of points with around −40 mg/kWh difference from the reference was due to the −6 ppm wrong quantification (interference) (discussed in Figure 3b). To put the results in context, the Euro VI limit is 460 mg/kWh for the combined cold start (weighted 14%) and hot start (weighted 86%) WHTCs. The limit could be achieved, for example, with emissions of 2000 mg/kWh and 200 mg/kWh for the cold and hot start WHTCs, respectively. The NO_x_ limit in California (USA) for 2024 is set at 0.05 g/bhp-h (67 mg/kWh) and will go down to 0.02 g/bhp-h in 2027 (27 mg/kWh). 

**Figure 5:**
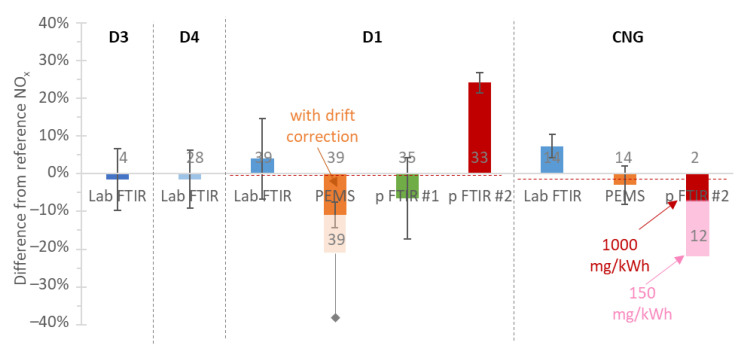
Comparisons of instruments measuring NO_x_ vs. laboratory CLD analyzers. Red dashed lines give the means of all the instrument measurements. Error bars are one standard deviation of the number of repetitions given in/on the bars. CLD = chemiluminescence detection; CNG = compressed natural gas engine; D = diesel engine; FTIR = Fourier transform infrared; p = portable; PEMS = portable emissions measurement system (with CLD analyzer).

The relative final emissions differences of each instrument from the laboratory reference CLD analyzers are summarized in Figure 5, separately for each engine. The mean differences were in most cases within ±10%, with some cases reaching ±25%. The 20% underestimations with PEMS and the portable FTIR #2 were explained in Figure 2b and Figure 3b, respectively (drift and interference offset). For engines D1 and CNG, many instruments were available and the mean value of all instruments was very close to the reference laboratory analyzers, as shown by the red dashed line.

### 3.2. CO_2_

**Figure 6:**
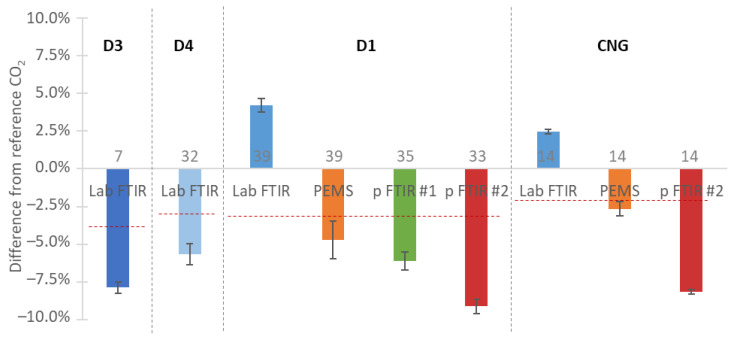
Comparisons of instruments measuring CO_2_ vs. laboratory NDIR analyzers. Red dashed lines give the means of all the instrument measurements. Error bars are one standard deviation of the number of repetitions given on/in the bars. CNG = compressed natural gas engine; D = diesel engine; FTIR = Fourier transform infrared; p = portable; PEMS = portable emissions measurement system (with heated NDIR analyzer).

Figure 6 plots the relative differences of each instrument from the reference laboratory NDIR analyzers, separately for each engine.

The mean differences were in most cases within ±7.5% for emissions that range from 560 to 785 g/kWh, with no trend in the function of emission levels. It should be mentioned that the reference NDIRs were the only analyzers that measured “dry” exhaust gas and they needed a dry-to-wet correction. Based on H_2_O measurements from the PEMS and the portable FTIR #2, this correction had an uncertainty of 2% or less for a complete cycle [42]. Assuming that there was no reference instrument and taking as a reference the mean of all instruments, the CO_2_ differences were within ±5% (see Figure 6, showing differences of instruments from the dashed line).

### 3.3. NH_3_

**Figure 7:**
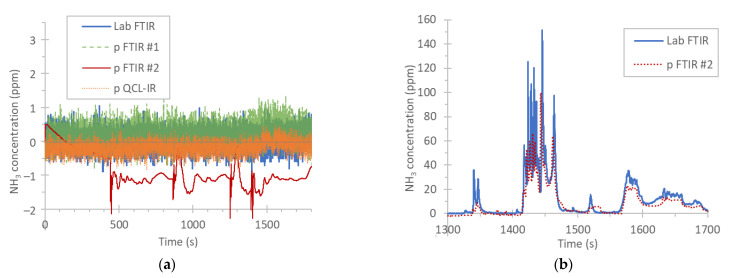
Real time NH_3_ concentrations: (**a**) hot start WHTC with the diesel engine D1; (**b**) part of a WHTC with the CNG engine. CNG = compressed natural gas engine; FTIR = Fourier transform infrared; p = portable; PEMS = portable emissions measurement system; QCL-IR = quantum cascade laser infrared; WHTC = world harmonized transient cycle.

Figure 7a plots the NH_3_ emissions of a diesel engine over a hot WHTC. The NH_3_ concentration was practically zero ppm throughout the test. The mean concentrations ranged from −0.9 ppm to 0.1 ppm. This graph and the respective values are a good indication of the background and zero levels of the instruments that can result even with negative values (still low).

Figure 7b plots the NH_3_ emissions of a CNG engine over a part of a WHTC. The two systems that were available had a difference of 30% for NH_3_ emission levels of 20 mg/kWh (or ±15% with respect to their mean value). The mean cycle concentration was 4 ppm (note that the limit is 10 ppm mean cycle concentration). The portable FTIR measurements were lower throughout the cycle. Although part of the difference could be a calibration issue, NH_3_ is sensitive to condensation and water interference, so the setup might have also contributed. Differences in the rise time might have also contributed.

### 3.4. N_2_O

**Figure 8:**
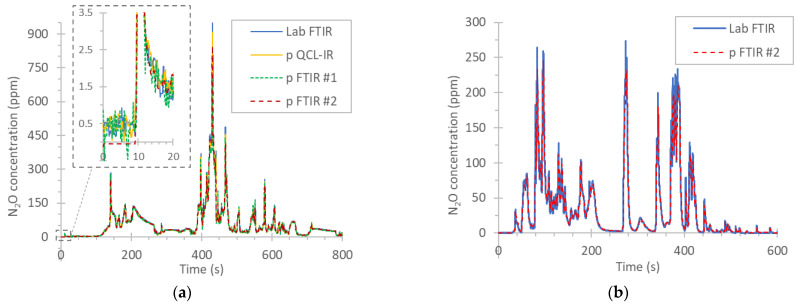
Real time N_2_O concentrations over the first minutes of cold start WHTCs: (**a**) diesel engine D1; (**b**) CNG engine. CNG = compressed natural gas engine; FTIR = Fourier transform infrared; p = portable; PEMS = portable emissions measurement system; QCL-IR = quantum cascade laser infrared; WHTC = world harmonized transient cycle.

Figure 8a plots 800 s of a cold start WHTC with the engine D1. The concentrations reached up to 900 ppm, and the emissions of the whole cycle were 185 mg/kWh. The differences of the instruments were <10%. The inset of the figure plots the first 20 s. The agreement of the instruments was very good even in the 5 ppm range, with background levels between 0 and 0.5 ppm. This background could be a small zero offset of the instruments or the true background concentration of N_2_O in the tubing [43].

Figure 8b plots the first 600 s of a cold start WHTC with the CNG engine. Only two instruments were available. The peaks reached 270 ppm, and the difference of the two instruments was <2% (at an emission level of 60 mg/kWh). Although not shown in the figure, the zero levels were around 0 ppm (laboratory FTIR) and 0.5 ppm (portable FTIR #2).

**Figure 9:**
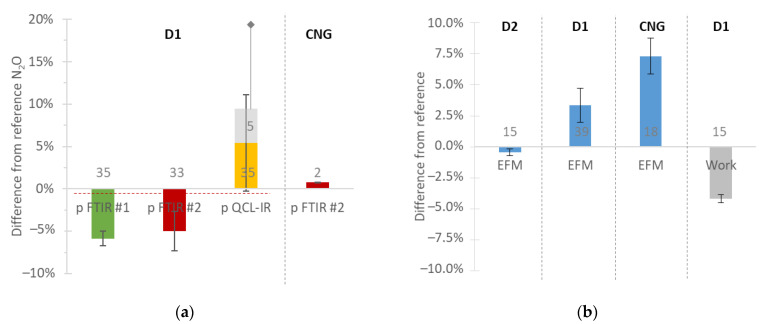
Comparisons of instruments: (**a**) N_2_O; (**b**) flow and work. Error bars are one standard deviation of the number of repetitions given in/on the bars. CNG = compressed natural gas engine; D = diesel engine; EFM = exhaust flow meter; FTIR = Fourier transform infrared; p = portable; PEMS = portable emissions measurement system; QCL-IR = quantum cascade laser infrared.

Figure 9a summarizes the available results, with instruments measuring N_2_O. The mean differences were within ±5% for emissions ranging between 30 mg/kWh and 190 mg/kWh. The laboratory FTIR was considered as the reference. Even when considering the error bars, the differences were within ±10%. The only exceptions were five tests which had an offset of 1 ppm, probably due to wrong calibration, resulting in a difference of 20%. To put the results into context, in the USA, the N_2_O limit is 0.10 g/bhp-h (134 mg/kWh).

### 3.5. Exhaust Flow and Work

Figure 9b summarizes the relative differences of three exhaust flow meters from the exhaust flow rate calculated by the engine dynamometer (fuel and intake air). The differences were up 7.5% (with the CNG engine). The mean exhaust flow rates were 220 kg/h for the CNG engine and 450 kg/h to 650 kg/h for the diesel engines.

Figure 9b also shows the differences in work as calculated by the OBD and the engine dynamometer for one case. The mean difference was around 5% for works ranging from 25 kWh to 250 kWh.

## 4. Discussion

The future Euro VII limits will be based on the performance of vehicles on the road. This means that they will have to include the measurement uncertainty of portable equipment. The current Euro VI and previous standards were based on laboratory measurements. For on-road ISC tests, which were introduced with Euro VI, a conformity factor is applicable to take into account the additional measurement uncertainty of the on-board equipment (PEMS) compared to the laboratory-grade equipment. For regulated pollutants (e.g., NO_x_), this factor is 1.5 (i.e., 50% additional measurement uncertainty) [22,24]. For NH_3_, no on-road measurement is required.

Uncertainty can be estimated by combining [22]:

- The uncertainty of the components that are needed for the calculation of the emissions (i.e., analyzer, exhaust flow, work) (see Equation (1));
- The uncertainty of the drift of the analyzers;
- The uncertainty of second-by-second measurements (dynamicity, time alignment);
- The impact of the boundary conditions (ambient temperature, altitude) on the instrument’s response.

Additionally, in our study, potential uncertainty related to the measurement technics applied should be considered. For light-duty vehicles, the NO_x_ conformity factor from 50% was reduced to 43%, and subsequent reports recommended a further decrease to 32% or 23%, or even 10% with drift correction and further restrictions of the drift and the validations of the PEMS in the laboratory [44]. For heavy-duty vehicles, the NO_x_ conformity factor is 50%.

The impact of boundary conditions for heavy-duty applications is often small because the vibrations are minimal and in a few cases a PEMS can be installed in temperature-controlled trailers. The dynamicity and time misalignment is usually <5%, as most studies have shown [23]. This leaves the uncertainties of the main components as the most significant contributing factors, which were the focus of this paper. The work uncertainty was found to be 5% (a maximum of 10% allowed by the regulations). The exhaust flow uncertainty was up 7.5%, which is in line with estimations from heavy-duty [45] and light-duty vehicles [44].

The first pollutant that needs to be discussed is NH_3_. It is already regulated, but no ISC tests on the road have been conducted. The data collected in our study were limited because under all conditions examined the NH_3_ slip of the diesel aftertreatment was negligible. The NH_3_ emissions of the CNG engine were also low, reaching <40% of the 10 ppm limit only at the cold start cycles (equivalent to 20 mg/kWh). The difference of the portable FTIR #2 was 30% compared to the laboratory FTIR, which is higher than the 3% prescribed in the regulation (or the difference was ±15% from the mean value). Although in the specific case the main reason was the underestimation of the concentrations, a 2 ppm zero offset, which is still within the regulation requirements, could easily result in 50% differences (for 4 ppm mean emissions). For the specific tests, it is highly likely that the setup contributed to the 30% differences because typically differences of up to 10% are expected [26,33,36,46,47]. NH_3_ can be easily “lost” when water condensation takes place (e.g., in the tubes until the sampling point), so the position of the instruments plays an important role [48]. Inadequate spectral resolution can result in large differences [36], though this was not the case for the FTIRs in our study.

The other pollutant that is under discussion is N_2_O. The agreement of the instruments was well within 10% for emission levels 30–190 mg/kWh. The zero (or background) levels were approximately 0.5 ppm. The results of this study confirmed that N_2_O can be measured on-road with relatively small uncertainty, in agreement with the results of others [35,36,43,49].

Another topic under discussion is the use of FTIR to measure regulated pollutants currently measured by a PEMS. The reason is that if FTIR is used to measure NH_3_ and N_2_O, then using the FTIR for the rest of the regulated pollutants would remove the need for installation of a PEMS. FTIRs practically do not drift and do not need regular calibration [26]. The results showed that the CO_2_ was within ±7.5% of the reference. However, the reference used a “dry” measurement (i.e., removal of the water and a dry-to-wet correction) [42]. When the instruments were compared to the mean of all instruments, the differences were within ±5%. Such levels are commonly reported in the literature [26,32,50,51]. The PEMS seemed to have a smaller difference from the reference compared to the portable FTIRs, although the data are not sufficient to confirm this. The PEMS used the same principle (NDIR) as the reference but measured “wet” exhaust (heated NDIR) instead of “dry”.

Finally, NO_x_ was examined. At emissions >200 mg/kWh, the differences were within ±10%, in agreement with a FTIR review [26]. At lower levels (<200 mg/kWh), the majority of the differences were within ±20 mg/kWh. Based on a limited number of tests, below 40 mg/kWh, the differences were within 15 mg/kWh. There were a few exceptions though: at 150 mg/kWh (CNG engine), the portable FTIR #2 was underestimating by 25% due to a −6 ppm zero error, probably due to water interference. At 50–100 mg/kWh emission levels, the PEMS was underestimating by 20 mg/kWh due to a −1 ppm zero drift. At 70–100 mg/kWh emission levels, the portable FTIR #2 was overestimating by 25–30 mg/kWh due to a 2 ppm zero offset.

**Figure 10:**
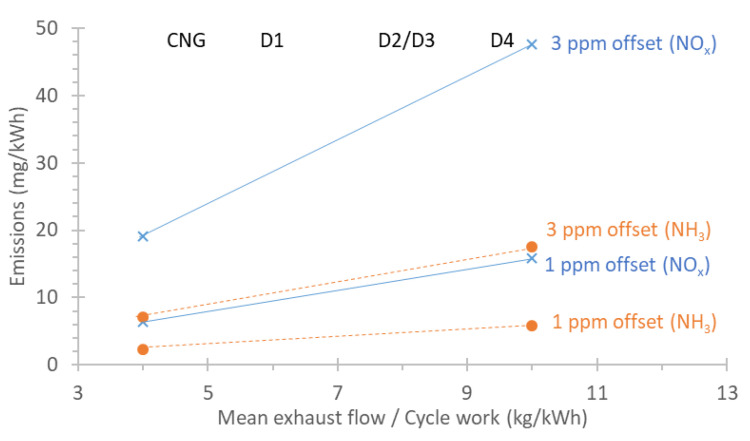
Zero levels and minimum detection limits. CNG = compressed natural gas engine; D = diesel engine.

In order to better understand the impact of the zero levels on the measurable levels of the portable instruments, the following equation was applied [52]:

(2) $$E_{gas,zero}=u_{gas}\left(\frac{Q}{W}\right)c_{zero}$$

where *E_gas,zero_* (mg/kWh) is the emissions measurable level, *c_zero_* (ppm) the zero offset (or background), and *Q*/*W* (kg/kWh) is the ratio of mean exhaust flow and the cycle work. For a diesel engine, *u_gas_* is 0.001586 for NO_x_ and 0.000586 for NH_3_. N_2_O, which has almost the same molar mass as NO_x_ (44 vs. 46 g/mol), has a *u_gas_* of 0.001517, which is close to that of NO_x_ (Table 5 of UNECE Regulation 49). Figure 10 translates a zero offset (or uncertainty) (in ppm) to emissions (in mg/kWh) for NO_x_ and NH_3_. For the x-axis, the ratio of the mean exhaust flow to the cycle work was used. This ratio ranged from 4.5 (CNG engine) to 9 (D4) for the cycles of this study (WHTC WHSC, ISC-like). The following conclusion can be drawn:

- A 1 ppm, NO_x_ zero offset translates to 16 mg/kWh offset for a large ratio (10 kg/kWh), but 6 mg/kWh for a small ratio (4 kg/kWh);
- Higher offsets result in higher detection limits (proportionally). As a worst case (10 kg/kWh), a 3 ppm NO_x_ zero offset is almost 50 mg/kWh offset;
- For NH_3_, the detection levels are almost three times lower due to the three times lower *u_gas_*.

The results of our study showed that for N_2_O a <0.5 ppm zero offset is possible, for NH_3_ a <1 ppm zero offset, and for NO_x_ a <2 ppm zero offset. For NO_x_, however, a higher value was noticed with one portable FTIR #2 with the CNG engine. This was attributed to water interference. As all FTIRs consisted of similar parts, our assumption is that the deconvolution of the spectra played a significant role. This highlights the need for strict technical requirements: one proposal is to require annual zero and span checks with “wet” gas (i.e., cylinders combined with water content), as required in the European Committee for Standardization (CEN) standards for PEMS performance assessment [53]. This high offset also showed that the laboratory “dry” detection limit might not always be representative of real applications.

## 5. Conclusions

The comparison of portable systems with laboratory-grade equipment showed that the agreement of the instruments was around ±5% for CO_2_, ±10% for NO_x_ or ±20 mg/kWh (whichever was larger), ±10% for N_2_O, and ±15% for NH_3_ at a wide range of emissions. The testing also revealed that a wide range of principles can be used to measure various exhaust gas compounds without significantly compromising the uncertainty. However, for future low emission levels from engines and the low limits set by regulations, particular attention needs to be paid to zero levels, which could contribute 10–30 mg/kWh to final emission results. It is recommended to further restrict the technical requirements and/or ensure that any specifications are fulfilled under realistic exhaust gas concentrations.

## Figures and Tables

**Table 1:** Overview of tested parameters and the equipment of the various engines. Note that different portable and laboratory instruments were used with each engine (except for p FTIR #2).

System	Portable	D1	D2	D3	D4	CNG
Lab analyzers	N	Y	Y	Y	Y	Y
Work (OBD)	Y	N	Y	N	N	N
EFM	Y	Y	Y	N	N	Y
PEMS	Y	Y	Y	N	N	Y
Lab FTIR	N	Y	N	Y	Y	Y
p FTIR #1	Y	Y	N	N	N	N
p FTIR #2	Y	Y	N	N	N	Y
p QCL-IR	Y	Y	N	N	N	N

CNG = compressed natural gas; D = diesel engine; EFM = exhaust flow meter; FTIR = Fourier transform infrared; p = portable; PEMS = portable emissions measurement system; QCL-IR = quantum cascade laser infrared.

**Table 2:** Technical specifications of the instruments. The only principle that needs removal of water from the exhaust gas (dry measurement) is NDIR. Heated NDIR, CLD, FTIR, and QCL-IR measure “wet” exhaust.

Requirement	Lab Analyzers	Lab Analyzers	PEMS	Lab FTIR	p FTIR #1	p FTIR #2	p QCL-IR
Manufacturer	AVL	Horiba	Horiba	AVL	IAG	A&D	Horiba
Model	AMA i60	MEXA-ONE	OBS-ONE	Sesam i60	OFS	BOB-1000FT	OBS-ONE-XL
CO_2_	NDIR	NDIR	Heated NDIR	Yes	Yes	Yes	-
NO_x_	CLD	CLD	CLD	Yes	Yes	Yes	-
N_2_O	-	-	-	Yes	Yes	Yes	Yes
NH_3_	-	-	-	Yes	Yes	Yes	Yes
Sampling line	6 m (191 °C)	6 m (191 °C)	6 m (191 °C)	6 m (191 °C)	6 m (191 °C)	6 m (191 °C)	6 m (113 °C)
t_10–90_	≤2.5 s	≤2.5 s	≤3.0 s	≤3.0 s	≤1.0 s	≤2.0 s	≤2.0 s
Qs (L/min)	13	13	3	8	10	10	3.3

Only those assessed in this study. CLD = chemiluminescence detection; FS = full scale; FTIR = Fourier transform infrared; NDIR = non-dispersive infrared; p = portable; PEMS = portable emissions measurement system; QCL-IR = quantum cascade laser–infrared; Qs = sampling flow rate.

## Data Availability

Data available from the corresponding author upon request.

## Appendix A

The comparison of the instruments was based on concentrations (ppm) or mass emissions (mg/kWh) using the same flow rate to reduce the impact of the exhaust flow rate. Table A1 gives an example with the results of various instruments for a part of a cycle where the emissions were very low, a part of the cycle where the emissions were high, and the complete cycle (WHTC). The differences of the instruments compared to the reference are given as percentages referring to concentration (ppm) differences or mass emission (mg/kWh) differences. For laboratory FTIR, the differences using concentrations or mass rates were similar (low: 2–4%, high 7%). Small differences were noticed between low and high emissions (2% vs. 7%) due to the small differences in the background levels of the two instruments. For the PEMS, the differences in concentrations and emission rates were close, but there was an effect. The reason is that the concertation differences weight every second equally, while the emission rates give more weight to those seconds in which the exhaust flow rate is high. Thus, the effect of the background was smaller. In this case, the PEMS had a −1 ppm offset. While at high concentrations the difference from the reference was −8%, at low concentrations it was −63%. With correction of this offset by 0.5 ppm (as allowed by the regulations), the deviations to the reference decreased.

Table A2 continues the example with other instruments. Both the portable FTIR #1 and #2 had relatively similar differences from the reference using concentrations or emission rates at both low and high concentrations. The impact of time misalignment is demonstrated with the portable FTIR #1 (±0.6 s). The differences change ±2%. This variability is what is usually expected [23], but higher differences have been reported depending on how dynamic the signals are (in terms of response time of the instrument and dynamicity of the cycle). The table also gives the results with a smoothened signal for the portable FTIR #1 (using moving average of 3 s). While the spikes were smoothened, the mean differences from the reference remained relatively the same. The response of the instruments is an important and difficult topic [54], but at tailpipe conditions with instruments having response times of 2 s (±1 s) the impact is relatively small for typical type approval cycles.

**Table A1:** NO_x_ emissions of a WHTC separated at a part with low emissions, high emissions, or the complete cycle, as determined with the reference CLD. Differences of other instruments to the reference using average concentrations (ppm) or emission rates (mg/kWh).

Cycle	Ref CLD		Lab FTIR		PEMS		PEMS (Drift Corr.)	
Part	(ppm)	(mg/kWh)	(%)_ppm_	(%)_mg/kWh_	(%)_ppm_	(%)_mg/kWh_	(%)_ppm_	(%)_mg/kWh_
Low	2.5	16.5	2%	4%	−63%	−51%	−43%	−33%
High	14.0	48.6	7%	7%	−8%	−8%	−5%	−5%
WHTC	4.6	70.8	5%	6%	−33%	−20%	−22%	−13%

CLD = chemiluminescence detection; FTIR = Fourier transform infrared; p = portable; PEMS = portable emissions measurement system; WHTC = world harmonized transient cycle.

**Table A2:** NO_x_ emissions of a WHTC separated at a part with low emissions, high emissions, or the complete cycle, as determined with the reference CLD. Differences of other instruments to the reference using average concentrations (ppm) or emission rates (mg/kWh).

Cycle	Ref CLD	p FTIR #2		p FTIR #1		+0.6 s	−0.6 s	Smooth 3 s
Part	(mg/kWh)	(%)_ppm_	(%)_mg/kWh_	(%)_ppm_	(%)_mg/kWh_	(%)_mg/kWh_	(%)_mg/kWh_	(%)_mg/kWh_
Low	16.5	12%	20%	−7%	−13%	−15%	−11%	−13%
High	48.6	13%	14%	−1%	−2%	−4%	0%	−2%
WHTC	70.8	14%	17%	−4%	−5%	−8%	−4%	−6%

CLD = chemiluminescence detection; FTIR = Fourier transform infrared; p = portable; PEMS = portable emissions measurement system; WHTC = world harmonized transient cycle.
